# Case Report: Gastro-bronchial fistula complicating a sleeve gastrectomy: from a glimmer of hope to a plight

**DOI:** 10.12688/f1000research.169504.2

**Published:** 2025-12-08

**Authors:** Adala Mourad, Adala Ahmed, Siala Rakia, Mseddi Mohamed Ali, Yaakoubi Chaima, Ben Radhia Bechir, Amara Amal, Sallemi Karim, Guizeni Rami, Ghariani Brahim, Sassi Karim, Ben Slima Mohamed

**Affiliations:** 1 Tunis obesity and diabetes surgery center, Tunis, Tunisia; 2 general surgery "B", Rabta Hospital, Tunis, Tunis, Tunisia

**Keywords:** sleeve gastrectomy, gastrobronchiol fistula, bariatric surgery

## Abstract

**Background:**

Bariatric surgery, particularly sleeve gastrectomy (SG), has emerged as an effective long-term treatment for morbid obesity. Despite its benefits, howver, it may result in severe complications. One rare but serious postoperative issue is the development of a gastrobronchial fistula (GBF), a condition with a challenging diagnosis and management pathway due to its insidious nature.

**Case Presentation:**

We report the case of a 36-year-old woman who underwent sleeve gastrectomy in 2015. The early postoperative course was complicated by a gastric fistula that was managed with double pigtail stents. Subsequently, the patient developed recurrent bronchopulmonary infections, and imaging in 2017 revealed a GBF connecting the gastric remnant to the bronchial tree. Initial endoscopic management with stenting failed because of migration. Definitive surgical management involved complex adhesiolysis and creation of tension-free fistula-jejunal anastomosis. Postoperative recovery was uneventful, and the patient remains asymptomatic.

**Discussion:**

Gastrothoracic fistula post-bariatric surgery is a rare but potentially life-threatening complication. Their development is often linked to the insufficient treatment of early gastric leaks or collections. Diagnosis is frequently delayed owing to nonspecific respiratory symptoms. Endoscopic approaches have show limited success, and surgical management, often complex, is frequently necessary. Multidisciplinary strategies, including endoscopic and surgical options, are vital for achieving favorable outcomes.

**Conclusion:**

Gastrobronchial fistulas represent a diagnostic and therapeutic challenge following sleeve gastrectomy. A high index of suspicion, long-term follow-up, and tailored multidisciplinary approach are essential for effective management and resolution. Awareness of this rare complication should prompt early detection and intervention to reduce the morbidity and mortality.

**Abbreviations**
AE: adverse events
BMI: body to mass index
CT: computed tomography
EOGD: esophagogastroduodenoscopy
GBF: gastrobronchial fistula
POD: postoperative day
SG: sleeve gastrectomy

## Introduction

Obesity is common. It affected 1 in 8 persons wordwide, according to the World Health Organization, it affects one in eight persons wordwide in 2022. ^1^ A review of data from the Global Burden of Disease registry in 2021, showcased increased by 2,5 fold in death and disability-adjusted life years attributable to high weight. ^2^ There is great disparity in prevalence on an international scale. ^3^ The low to middle socio-demographic index experienced the highest annual percentage increase in age standardized deaths and disability-adjusted life-year rates caused by obesity. ^2^ A meta-analysis of two decades of World Health Organization reports concluded an association and a 14% increase in the chance of obesity with increasing economic status. ^3^ For these reasons, decision makers and the medical community have suggested bariatric surgery as a secure and durable cure for obesity in selected patients. ^4^ The American Society for Metabolic and Bariatric surgery estimated 279967 national inpatient surgeries by 2022. ^5^ This enthousiasm and rising trend in performing bariatric surgery is justified by 29%, 43%, and 72% of cardiovascular disease, cancer, and diabetes rates dropping after surgery. ^6^ Sleeve gastrectomy outnumbered other surgical and endoluminal interventions in the latest report by the International Federation for the Surgery of Obesity and Metabolic disorders in 2024. ^7^

However, bariatric surgery can be fraught with severe post-operative complications. The National Institute of Diabetes and Digestive and Kidney Diseases disclosed 0,2% mortality rate for laparoscopic gastric bypass. ^8^ With open procedures, deaths reached 2,1%. ^8^ The mortality rate following sleeve gastrectomy was 0,22%. ^9^ A large propensity-matched comparison of 30-day morbidity and mortality of sleeve gastrectomy, Roux-en-Y gastric bypass, and one-anastomosis gastric bypass showed no significant difference in 30-day morbidity and mortality. ^10^ Although nonsurgical causes are the main cause of death, ^11^ surgery-related complications can cause postoperative mortality or complications.

Gastrobronchial fistula is an unseen post-sleeve gastrectomy complication. This poses both diagnostic and therapeutic challenges. It is insidious and can go unnoticed for several years, as was the case in our patient. We report a similar case that posed difficulties in its diagnosis and treatment. We aimed to shed light on its mechanisms to present a clear diagnosis and treatment plan.

## Case report

Here, we report the case of a 36 year old woman with no relevant medical history. She had a BMI of 42 kg/m ^2^ and required sleeve gastrectomy in 2015 in another surgical department. She denied smoking or drug use. The postoperative course was fraught with a gastric fistula, on 13 ^th^ postoperative day (POD). This complication was successfully managed with double-pigtail stenting for 8 weeks.

One year after the index surgery in 2016, she underwent cholecystectomy for de novo cholecystolithiasis. On the 6 ^th^ POD, purulent left subphrenic collection was diagnosed after she experienced left upper quadrant pain and fever. Percutaneous drainage was warranted with a good response.

In 2017, she reported cough with recurrent bronchopneumonia. Her weight loss was stable and had a BMI of 22 kg/mm ^2^. Given her worsening condition, a CT scan was ordered. It revealed a left subphrenic collection, atelectasis of the left lower pulmonary base, and a fistulous tract between the gastric remnant and the ronchial tree. On EGD, a large fistula measuring 10 mm was discovered in the cardia. Our patient had persistent stable respiratory status. The multidisciplinary decision was to stent the fistulous tract, which failed three weeks after prosthesis 3 weeks later.

**Figure 1.**
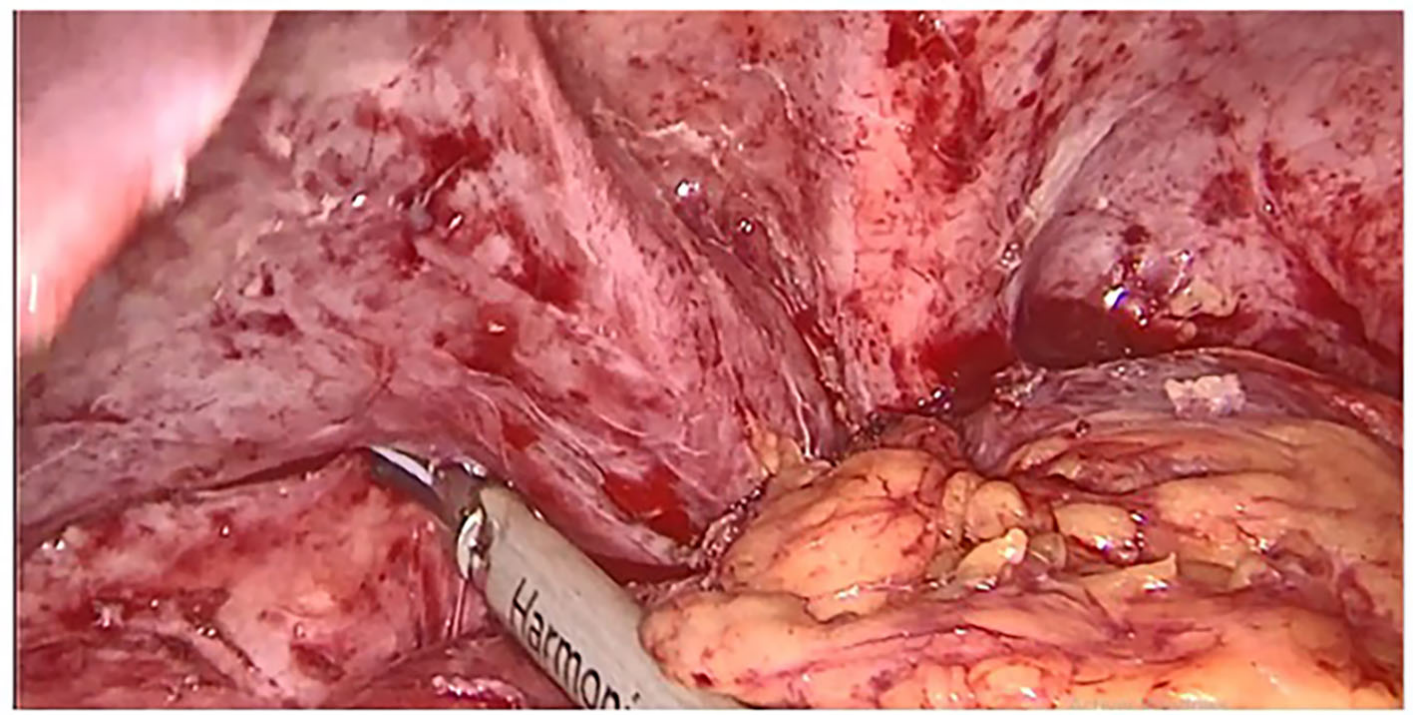
Adhesiolysis of peri fistular fibrous adhesions.

**Figure 2.**
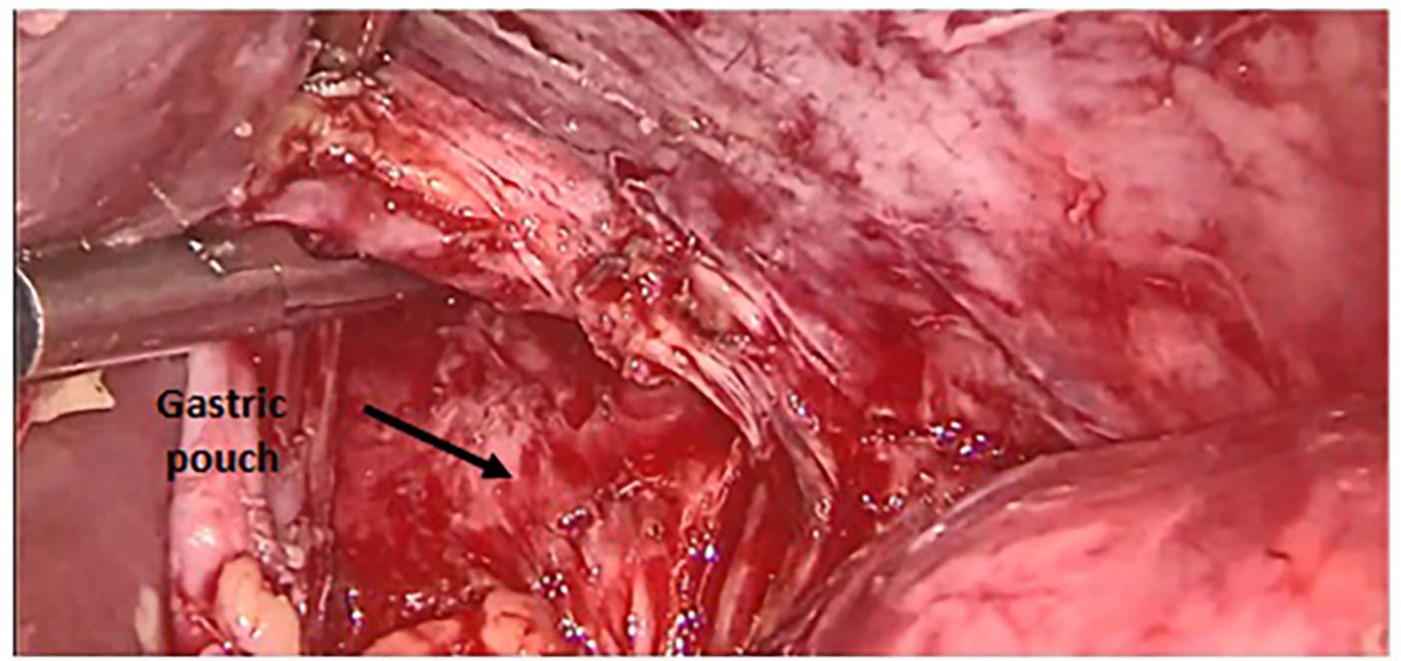
Intra thoracic dissection of gastric pouch.

**Figure 3.**
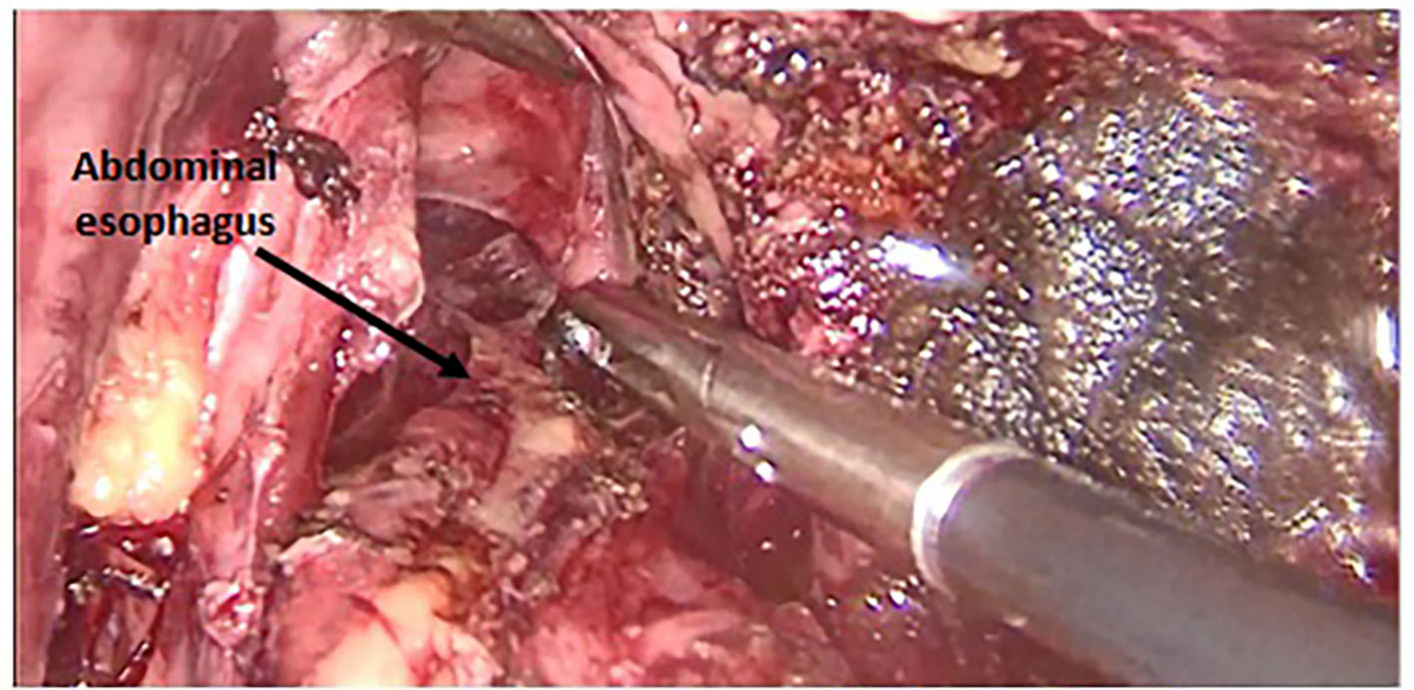
Lowering the mediastinal esophagus.

**Figure 4.**
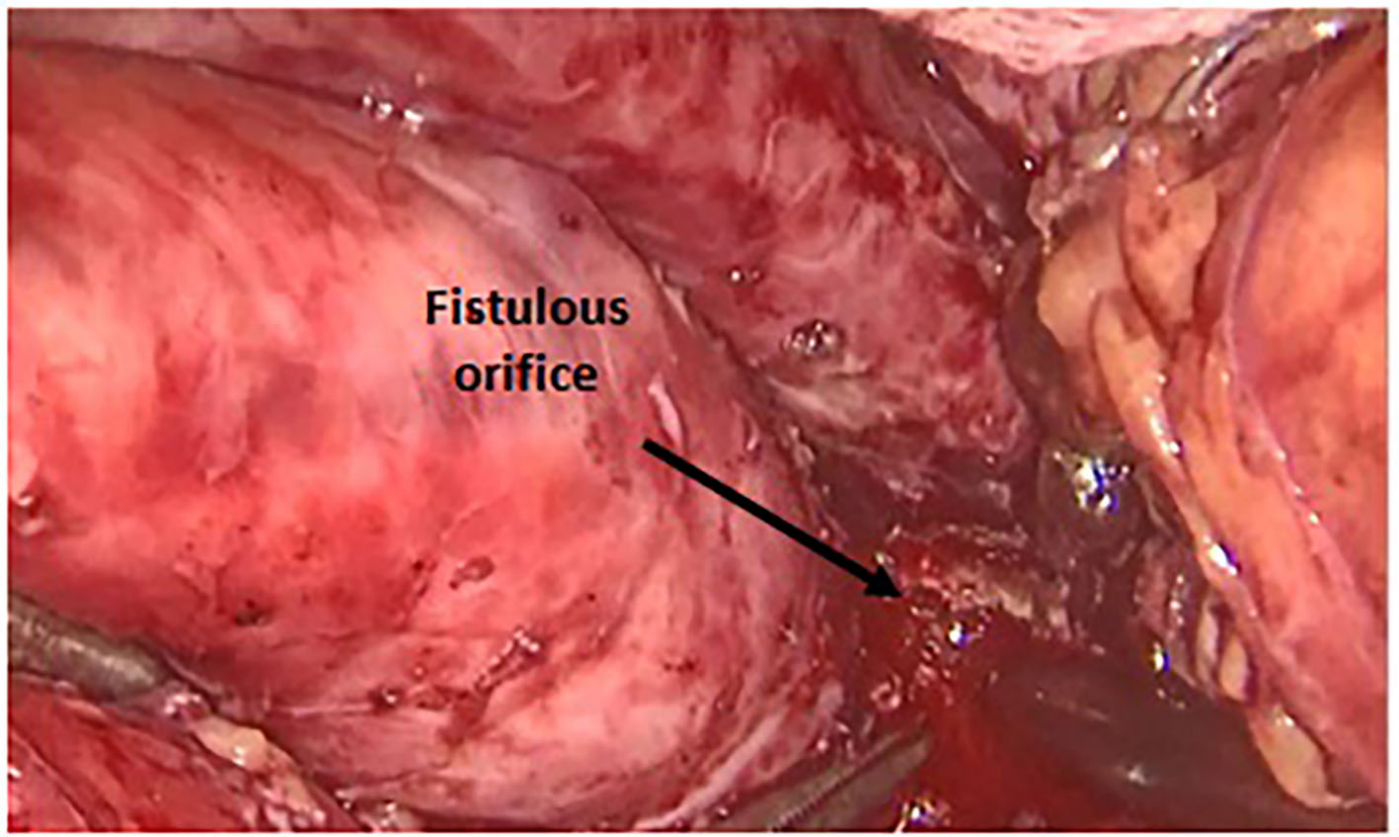
Operative view of the fistulous orifice within the gastric wall.

**Figure 5.**
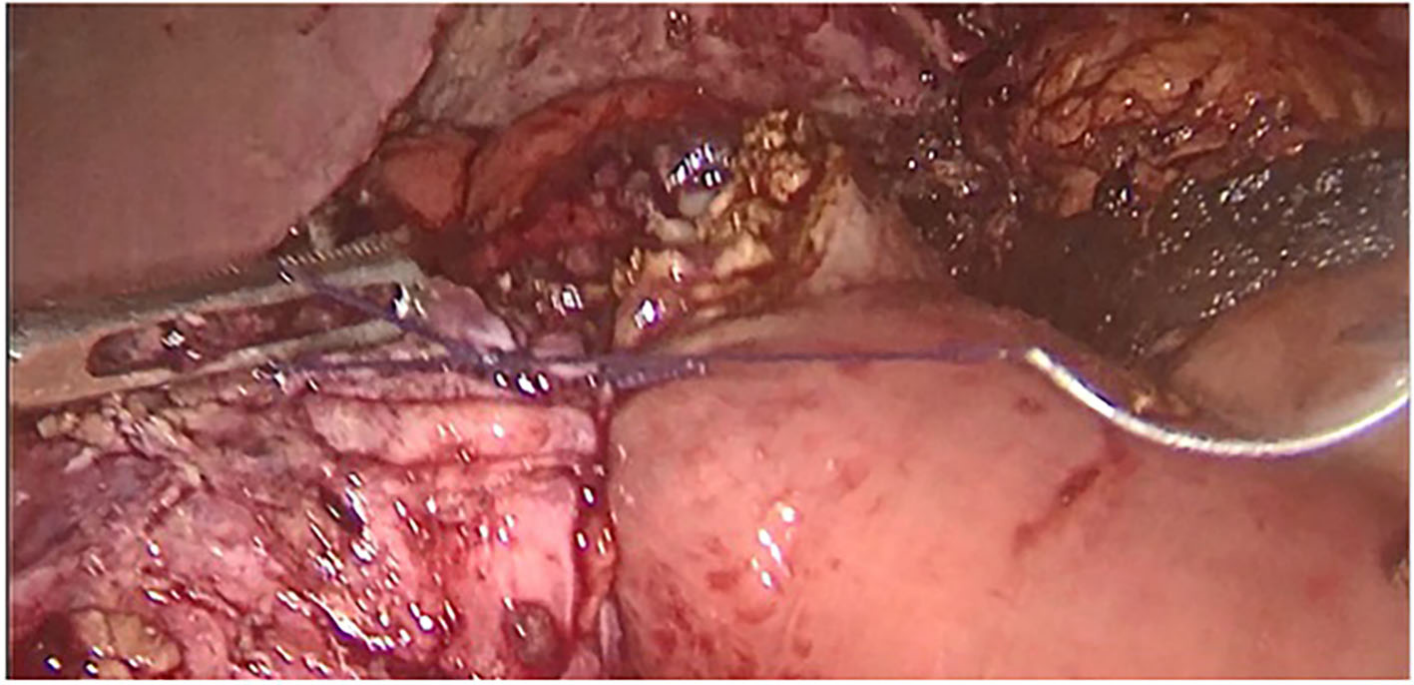
Roux-en-y fistulo-jejunostomy.

Surgery was then performed. After pneumoperitoneum was established using Hasson technique, dense adhesions between the gastric tube, the left hepatic lobe, and the left diaphragm were freed; using scissors to avoid energy related injuries. Peri-fistular fibrosclerosis made the dissection taut and hemorrhagic ( Figure 1). There was a fistulous tract on the anterior aspect of the gastric pouch measuring 1 cm in diameter with hardened edges. To achieve tension-free fistula-jejunal anastomosis, the intra thoracic esophagus was freed ( Figures 2 and 3). A jejunal loop, 60 cm from the ligament of Treitz, was raised and anastomosed manually in a termino-lateral fashion; with a running 4-0 vicryl thread ( Figures 4 and 5). Surgical intervention was completed by drainage of the hiatal orifice. Our patient was discharged on the 10 ^th^ POD and has remained well since then. Five years later, the patient did not report dysphagia or cough, and had a stable BMI.

## Discussion

Gastrothoracic fistulas are serious complications that are relatively rarely described after bariatric surgery, for which there is no consensus on management.

These have been described following sleeve gastrectomy or gastric bypass. A multicenter French cohort included 24 cases from 2007 to 2018, of which 21 underwent sleeve gastrectomy and 3 underwent gastric bypass. ^12^ Sometimes, it is not the result of a gastric fistula, but rather the aftermath of inappropriate treatment of the latter. According to a review of 76 patients with intra thoracic gastric fistula, a history of gastric fistula was made in 57,5% of cases. ^13^ And in 26,25% of the cases, patients were insufficiently treated for abdominal or mediastinal collection. ^13^ Inappropriate stent size, position, stenting duration, and persistence of low-grade inflammation could explain the reported case. ^14^ The development of the broncho-gastric fistula in our case can be understood as a sequential process in which an early proximal staple-line leak gave rise to a persistent subphrenic collection. Over time, this collection evolved into a true abscess whose inflammatory and enzymatic activity caused progressive damage to adjacent tissues. The diaphragm, lying directly above the infected cavity, became exposed to continuous inflammation, local sepsis and ischemic stress, ultimately leading to its erosion and to the creation of a trans-diaphragmatic tract that extended toward the bronchial tree, establishing a broncho-gastric communication. This mechanism may be further exacerbated when a distal sleeve stenosis or functional obstruction is present, as increased intraluminal pressure promotes ongoing leakage, prevents adequate healing of the staple line, and sustains the subphrenic suppuration, thereby accelerating the erosion process and facilitating fistula formation. In fact, pus can eventually erode through the ipsilateral diaphragm, creating a pathological communication between the stomach, bronchial tree, or pleura, causing a gastro-bronchial or gastro-pleural fistula. ^15^ Downstream stenosis should be considered as chronic fistulas that are related to increased intraluminal pressure in the newly sized stomach. ^16^

The presentation is usually insidious. In a systematic review of 26 studies, respiratory symptoms were the most reported signs, with pneumonia taking the lead, next to subphrenic collections. ^17^ This is always a consequence of an anterior fistula. ^12^ A French study of 11 OGF cases concluded that in the majority of cases, the fistulous tract rises at the proximal end of the suture line. ^18^

Its treatment is not consensual and different attitudes have been suggested. However, given its complex nature, healing requires a long period. Fistula tract closure was achieved at an average of 7 months. ^12^ In one case, the healing time reached 7 years. ^17^ Endoscopic treatment, including clip or stent placement, has resulted in poor results. In fact, in a cohort of 24 cases, despite an average endoscopic treatment of 5 essays, 83% of patients underwent surgical procedures. ^12^ In another multicenter study, endoscopic internal drainage using stents was performed in 30 cases of gastro-bronchial fistulas and 10 gastrocolic fistulas following SG. ^19^ Despite the absence of major adverse effects, success was recorded in 47,5% of the cases. ^19^ This highlights the safety of endoscopic treatment for these complications despite their average results. In certain cases, when no endoscopic or surgical salvage procedures are efficient, total gastrectomy is performed. ^20^ In addition, a combined thoracic and abdominal route is often necessary foradequate debridement. This complication is often the cause of subsequent denutrtion (79%) explaining the high mortality rate reported in the literature (42%). ^12^ Finally, given the intense adhesion state, surgery can be limited to simple debridement, as stated in a recent review. ^13^

## Conclusion

Bariatric surgical procedures are constantly evolving with the increased necessity of radical treatment for obesity. Laparoscopic sleeve gastrectomy is the most commonly performed surgery, owing to its reproducibility and safety. However, this can lead to serious adverse events (AEs). It is crucial for surgeons to be aware of common and rare postoperative complications. The onset of GBF is challenging. Surgeons should trace patients postoperatively because their occurrence is subtle. Persistent or otherwise unexplained respiratory symptoms after SG should raise early suspicion for a GBF and prompt appropriate imaging. Chronic subphrenic sepsis, even when initially subtle, has the potential to erode the diaphragm and facilitate fistulization into the thoracic cavity, underscoring the need for timely drainage and source control. The treatment incorporates both endoscopic and surgical methods. Closure of the fistulous tract is possible because of the different strategies available. The choice of salvage plan should be tailored to the patient’s condition and the center’s expertise. A clear pivot point must also be established between prolonged endoscopic therapy and escalation to surgical rescue, particularly when sepsis persists, a mature fistulous tract becomes evident, or distal sleeve stenosis prevents durable healing. In such situations, fistulo-jejunostomy offers a rational and effective surgical option, as it diverts gastric flow away from the fistula, excludes the diseased segment, and enables definitive control of both the leak and the associated sepsis.

## Declarations

### Ethical approval

Not required. This work is a single case report, which is generally not considered “research” requiring formal review by an ethics committee. The case describes the clinical management of one patient without any experimental intervention or deviation from standard care.

## Consent

Written informed consent for publication of their clinical details and clinical images was obtained from the patient.

## Data Availability

*The project contains the following underlying data*: *10.5281/zenodo.16941530* . *Under the name: dataset for article “CARE checklist for manuscript 169504*” ^21^ Data are available under the terms of the Creative Commons Attribution 4.0 International license (CC-BY 4.0).
