# Supplementary material for: Case Report: Gastro-bronchial fistula complicating a sleeve gastrectomy: from a glimmer of hope to a plight
Source: F1000Res. 2025 Dec 8;14:948. Originally published 2025 Sep 19. [Version 2] doi: 10.12688/f1000research.169504.2 (PMC12754367; doi:10.12688/f1000research.169504.2)
Supplement: Peer review history

# Reviewer report: Reviewer response for version 2

**DOI:** 10.5256/f1000research.192320.r442107

**Published:** 2025-12-30

**Authors:** Kuo-Feng Hsu

**Affiliations:** 1 National Defense Medical University, Taipei, Taiwan

This manuscript presents a well-documented and clinically relevant case of a delayed gastro-bronchial fistula following sleeve gastrectomy. The authors describe the diagnostic challenge, failure of endoscopic management, and successful definitive surgical treatment with fistulo-jejunal anastomosis, achieving durable symptom resolution on long-term follow-up. The topic is important for bariatric and upper gastrointestinal surgeons, and the manuscript adds educational value by illustrating a complex salvage strategy for a rare but severe complication.

 The revised version has substantially improved in terms of pathophysiological explanation, operative rationale, and clinical clarity. Only **minor revisions** are suggested to further enhance completeness and transparency.

**Minor Comments / Points for Clarification**

1. **Clarification regarding total gastrectomy**
  In the Discussion, the authors appropriately mention that *total gastrectomy may be required in selected cases when endoscopic and surgical salvage procedures fail*. Given this statement and the severity of the present case, it would be important to explicitly clarify:
  - **Whether total gastrectomy was considered at any stage in this patient’s management**, and
  - If so, **why it was ultimately avoided in favor of fistulo-jejunostomy** (e.g., preserved gastric remnant viability, controlled sepsis, patient nutritional status, or desire to avoid the morbidity of total gastrectomy).
  A brief sentence addressing this decision-making process would significantly strengthen the surgical reasoning and help readers understand the indications and thresholds for escalating to total gastrectomy in similar scenarios.
2. **Surgical decision-making hierarchy**
  The authors may consider adding one short sentence in the Discussion summarizing their institutional or case-based algorithm (endoscopic → surgical diversion → total gastrectomy as last resort), reinforcing that fistulo-jejunostomy served as a definitive but organ-preserving alternative in this case.
3. **Minor language and formatting**
  Only minor language polishing is still needed in a few sentences (spacing, punctuation, and consistency of terminology), but these do not affect scientific content.

**Overall Assessment**

 This case report is scientifically sound, clinically instructive, and suitable for indexing after **minor revision**. Addressing the point regarding consideration of **total gastrectomy** will further improve the manuscript’s completeness and value for practicing surgeons.

Are enough details provided of any physical examination and diagnostic tests, treatment given and outcomes?

Yes

Is the case presented with sufficient detail to be useful for other practitioners?

Yes

Is sufficient discussion included of the importance of the findings and their relevance to future understanding of disease processes, diagnosis or treatment?

Yes

Is the background of the case’s history and progression described in sufficient detail?

Yes

Reviewer Expertise:

BARIATRIC SURGERY

I confirm that I have read this submission and believe that I have an appropriate level of expertise to confirm that it is of an acceptable scientific standard, however I have significant reservations, as outlined above.

# Reviewer report: Reviewer response for version 2

**DOI:** 10.5256/f1000research.192320.r439637

**Published:** 2025-12-27

**Authors:** Mohamed Hany

**Affiliations:** 1 Alexandria University, Alexandria, Egypt

Unaddressed Issues :

 1.  Echoing Reviewer 1, I stated that the “authors should clearly articulate what this case adds to existing knowledge beyond documenting another instance of a known complication.” I noted that “The current manuscript does not appear to introduce a new diagnostic modality, a novel therapeutic technique, or a fundamentally different understanding of the pathophysiology of GBF.”

  As mentioned, the revised manuscript fails to provide a clear statement of its novel contributions. The case is presented as a clinical example, but there is no explicit discussion of what makes it unique or what new insights it provides to the field.

 .

 2. I requested more detailed clinical information, noting that the report “does not provide details of the findings” from the CT scan and that information on “pulmonary function tests or the severity of the recurrent bronchopulmonary infections” would be beneficial. The reviewer also noted that “The report mentions a CT scan revealed the GBF, but it does not provide details of the findings. Including images or a more detailed description of the CT and endoscopic findings would be highly beneficial.”

  The revised manuscript mentions CT findings but does not provide detailed descriptions or images. The text states: “a CT scan was ordered. It revealed a left subphrenic collection, atelactasis of the left lower pulmonary base, and a fistula tract between the gastric remnant and the bronchial tree.” However, no CT images are included, and there is no information regarding the patient’s pulmonary function tests or the severity of the recurrent bronchopulmonary infections beyond the statement that the patient “reported cough with recurrent bronchopneumonia.”

  Basic CT findings are mentioned, but detailed descriptions, images, and pulmonary function information are absent.

 3. I highlighted the need for objective follow-up data, including “absence of cough/aspiration, nutritional status (weight/BMI, albumin), need for PPIs, pulmonary function or imaging, and any recurrence or re-intervention at defined intervals (e.g., 3/6/12 months).” I specifically noted: ”‘Asymptomatic’ is insufficient for a fistula. Report objective outcomes: absence of cough/aspiration, nutritional status (weight/BMI, albumin), need for PPIs, pulmonary function or imaging, and any recurrence or re-intervention at defined intervals (e.g., 3/6/12 months).”

  The manuscript states, “Five years later, the patient did not report dysphagia or cough and had a stable BMI.” However, it provides no objective data on nutritional status beyond BMI, no information on PPI usage, no pulmonary imaging follow-up, and no detailed recurrence assessment. The follow-up duration is mentioned as five years, but there is no specification of formal follow-up intervals or objective clinical assessments at defined time points.

 - Follow-up duration (five years) and absence of dysphagia/cough are mentioned, but objective nutritional data, PPI usage, pulmonary imaging, and recurrence assessment are absent.

 4. I recommended that the “CARE checklist items should be integrated into the manuscript (timeline; patient perspective; diagnostic challenges; rationale for decisions; follow-up).” I noted: “You reference a Zenodo item titled ‘CARE checklist for manuscript 169504.’ Please integrate key CARE items into the manuscript (timeline; patient perspective; diagnostic challenges; rationale for decisions; follow-up). Verify that the Zenodo link is functional and clearly labeled in Data availability.”

  The “Data availability” section mentions “The project contains the following underlying data: 10.5281/zenodo.16941530. Under the name: dataset for article ‘CARE checklist for manuscript 169504’” and Reference 21 is listed as “Mseddi MA: CARE checklist for manuscript 169504. Zenodo. 2025. Publisher Full Text.” However, the CARE checklist items (timeline, patient perspective, diagnostic challenges, rationale for decisions, follow-up) have not been explicitly integrated into the manuscript narrative. While the manuscript does provide some of this information scattered throughout, it is not organized according to the CARE checklist framework.

 The CARE checklist is referenced and available, but its items are not explicitly integrated into the manuscript structure as requested.

 5. I found the cited “42% mortality in the literature” to be alarming and requested “careful citation, context (era, selection bias, septic shock cohorts), and comparison with more recent series.” I specifically stated: “The discussion mentions a 42% mortality in the literature; this is alarming and requires careful citation, context (era, selection bias, septic shock cohorts), and comparison with more recent series. Re-check the source and either justify or temper the claim.”

 The revised manuscript’s discussion section states: “This complication is often the cause of subsequent denutrtion (79%) explaining the high mortality rate reported in the literature (42%).” This statement is cited to Reference 12, which is Marie L’s 2019 thesis “Enquête sur les fistules gastro-thoraciques après chirurgie bariatrique.” While the reference is now properly cited, the manuscript does not provide the context requested by the reviewer. There is no discussion of the era from which this 42% mortality rate derives, no mention of potential selection bias, no discussion of whether this rate applies to septic shock cohorts specifically, and no comparison with more recent mortality data.

  The 42% mortality rate is now properly cited to Reference 12, but the contextual information and comparison with recent data are absent.

 6. I pointed out several minor issues, including non-sequential figure numbering, the need for a clearer timeline of events, the presence of vague statements like “pus can eventually erode through the peripheral diaphragm,” and the need for consistent terminology.

  The figures in the manuscript are numbered 1 through 5 and appear to be sequential in the text. However, the timeline of events is presented in narrative form rather than in a structured table or chronological summary as suggested. The manuscript contains the statement “pus can eventually erode through the ipsilateral diaphragm, creating a pathological communication between the stomach, bronchial tree, or pleura, causing a gastro-bronchial or gastro-pleural fistula.” While this is slightly more precise than the original vague statement, it still lacks the scientific rigor expected in a medical manuscript. Terminology appears to be generally consistent, with “gastro-bronchial fistula (GBF)” being used throughout.

 Figure numbering appears correct, terminology is consistent, but the timeline remains in narrative form, and some statements could be more scientifically precise.

Are enough details provided of any physical examination and diagnostic tests, treatment given and outcomes?

Partly

Is the case presented with sufficient detail to be useful for other practitioners?

No

Is sufficient discussion included of the importance of the findings and their relevance to future understanding of disease processes, diagnosis or treatment?

Partly

Is the background of the case’s history and progression described in sufficient detail?

Partly

Reviewer Expertise:

MBS

I confirm that I have read this submission and believe that I have an appropriate level of expertise to confirm that it is of an acceptable scientific standard, however I have significant reservations, as outlined above.

# Reviewer report: Reviewer response for version 2

**DOI:** 10.5256/f1000research.192320.r439636

**Published:** 2025-12-27

**Authors:** Vusal Aliyev

**Affiliations:** 1 General Surgery, Alibey Hospital, Istanbul, Turkey

The changes are acceptable.

Are enough details provided of any physical examination and diagnostic tests, treatment given and outcomes?

No

Is the case presented with sufficient detail to be useful for other practitioners?

No

Is sufficient discussion included of the importance of the findings and their relevance to future understanding of disease processes, diagnosis or treatment?

No

Is the background of the case’s history and progression described in sufficient detail?

No

Reviewer Expertise:

General Surgery

I confirm that I have read this submission and believe that I have an appropriate level of expertise to confirm that it is of an acceptable scientific standard.

# Reviewer report: Reviewer response for version 1

**DOI:** 10.5256/f1000research.186852.r423300

**Published:** 2025-11-20

**Authors:** Vusal Aliyev

**Affiliations:** 1 General Surgery, Alibey Hospital, Istanbul, Turkey

This case report presents a rare but clinically important complication following sleeve gastrectomy—gastro-bronchial fistula (GBF). The topic is relevant to bariatric and thoracic surgeons, and the authors describe a well-documented patient course. However, the manuscript would benefit from significant refinement in structure, detail, and language. There are repeated or redundant sentences, typographical inconsistencies, and limited novelty in presentation.

**Major Comments: **

1. The *Introduction* contains repeated and awkwardly phrased sentences, especially the opening statement:
  - “Obesity is rare. It affected 1 in 8 persons worldwide... it affects one in eight persons worldwide in 2022.”
    This duplication should be corrected and revised to a scientifically accurate statement such as:
    “Obesity is a major global health issue affecting approximately one in eight people worldwide (WHO, 2022).”
  - The paragraph mixes epidemiologic data, mortality rates, and surgical volume without clear flow. Consider restructuring to:
    1. Global burden of obesity
    2. Rationale for bariatric surgery
    3. Frequency and complications of sleeve gastrectomy
    4. Rarity of gastro-bronchial fistula
2. **Novelty and Significance**
  - GBF after sleeve gastrectomy has been documented in multiple systematic reviews and recent reports. The case offers no clearly novel diagnostic or therapeutic innovation. The authors should emphasize what differentiates this report — e.g., the combination of post-cholecystectomy abscess contributing to fistula formation or the long latency period.
3. **Case Description**
  - The surgical and diagnostic details are overly brief:
    - Include imaging (CT, endoscopy) descriptions or figures with annotations.
    - Specify the size, location, and relation of the fistulous tract.
    - Clarify intraoperative findings and rationale for fistulo-jejunal anastomosis.
    - Add follow-up duration (months/years) and outcomes such as pulmonary recovery or nutritional status.
  - Correct inconsistent abbreviations: EGOD → EGD, “ronchial” → bronchial.
4. **Discussion**
  - Strengthen causal reasoning: explain how early leak and subphrenic abscess led to diaphragmatic erosion and GBF formation.
  - The cited 42% mortality rate is likely from older literature; verify source and contextualize.
  - Provide a schematic explanation or flow of pathogenesis (e.g., leak → abscess → erosion → communication).
5. **Language and Style**
  - The tone is sometimes overly dramatic (e.g., “from a glimmer of hope to a plight”). A more neutral scientific style is preferable.
  - Numerous grammatical and typographical errors should be corrected (e.g., “howver,” “double-pig,” “foradequate”).
  - Use consistent terminology: “gastro-bronchial fistula (GBF)” throughout.
6. **Figures**
  - Current intraoperative photos need labeling, arrows, and context. Add pre- and post-treatment imaging if possible.
7. **References**
  - Several references are incomplete or non-standard (e.g., “Obesity and overweight. 2025” is not a proper citation).
  - Add recent key works such as:
    - Sakran et al., *Obes Surg* 2021;31:357-369
    - Rogalski et al., *Prz Gastroenterol* 2020;15:356-362
    - Campos et al., *Obes Surg* 2011;21:1339-1345

**Minor Comments**

- Standardize decimals (0.22%, not 0,22%).
- Correct organization names (IFSO = *International Federation for the Surgery of Obesity and Metabolic Disorders*).
- Define all abbreviations on first use (SG, GBF, BMI, etc.).
- Provide clear chronological timeline (2015–2025).
- Include “Learning points” at the end summarizing:
  1. Chronic cough after SG warrants GBF evaluation.
  2. Early leaks or abscesses may cause thoracic fistulae.
  3. Combined surgical and endoscopic management yields best outcomes.

Are enough details provided of any physical examination and diagnostic tests, treatment given and outcomes?

No

Is the case presented with sufficient detail to be useful for other practitioners?

No

Is sufficient discussion included of the importance of the findings and their relevance to future understanding of disease processes, diagnosis or treatment?

No

Is the background of the case’s history and progression described in sufficient detail?

No

Reviewer Expertise:

General Surgery

I confirm that I have read this submission and believe that I have an appropriate level of expertise to confirm that it is of an acceptable scientific standard, however I have significant reservations, as outlined above.

# Reviewer report: Reviewer response for version 1

**DOI:** 10.5256/f1000research.186852.r419810

**Published:** 2025-11-04

**Authors:** Mohamed Hany

**Affiliations:** 1 Alexandria University, Alexandria, Egypt

The manuscript by Mourad et al. presents a case report of a 36-year-old woman who developed a gastro-bronchial fistula (GBF) as a rare and delayed complication of a sleeve gastrectomy performed in 2015. The authors describe the patient’s clinical course, including the initial management of a gastric leak, the subsequent development of recurrent respiratory infections, and the eventual diagnosis of a GBF in 2017. The report details the failure of endoscopic stenting and the successful definitive management with a complex surgical procedure involving adhesiolysis and a tension-free fistula-jejunal anastomosis. The authors conclude that GBF is a challenging complication that requires a high index of suspicion and a multidisciplinary approach for successful management.

  Major Issues

 The manuscript addresses an important and rare complication of bariatric surgery. However, several major issues need to be addressed to improve the quality and impact of this report.

 1. The primary concern is the limited novelty of the case. While GBF is an uncommon complication, it is a well-documented phenomenon. A recent literature search reveals numerous case reports and systematic reviews on this topic. For instance, a comprehensive systematic review by Sakran et al. (2021), which the authors cite, has already analyzed 55 cases of gastric fistulas in the chest after sleeve gastrectomy, providing a thorough overview of diagnostic and treatment options [1]. More recent case reports from 2024 and 2025 also describe similar presentations and management strategies [2, 3]. The current manuscript does not appear to introduce a new diagnostic modality, a novel therapeutic technique, or a fundamentally different understanding of the pathophysiology of GBF. The authors should clearly articulate what this case adds to the existing body of knowledge beyond simply documenting another instance of a known complication.

 2. The clinical details provided are insufficient for a thorough understanding of the case. Key information is missing, which limits the educational value of the report. Specifically:

     •     The report mentions a CT scan revealed the GBF, but it does not provide details of the findings. Including images or a more detailed description of the CT and endoscopic findings would be highly beneficial.

     •    There is no information on the patient’s pulmonary function tests or the severity of the recurrent bronchopulmonary infections.

     •     The description of the definitive surgical procedure is too brief. A more detailed, step-by-step description of the operative technique, including the approach to adhesiolysis and the specifics of the fistula-jejunal anastomosis, would be valuable for other surgeons who may encounter this rare complication.

     •    The report states the patient remained well since then,” but the duration of follow-up is not specified. This is a critical piece of information for assessing the long-term success of the surgical intervention.

 3. **CARE checklist & data availability**

 You reference a Zenodo item titled “CARE checklist for manuscript 169504.” Please integrate key CARE items into the manuscript (timeline; patient perspective; diagnostic challenges; rationale for decisions; follow-up). Verify that the Zenodo link is functional and clearly labeled in **Data availability.**

 4. The discussion mentions a **42% mortality** in the literature; this is alarming and requires careful citation, context (era, selection bias, septic shock cohorts), and comparison with more recent series. Re-check the source and either justify or temper the claim.  

 5. “Asymptomatic” is insufficient for a late thoraco-abdominal fistula. Report **objective outcomes**: absence of cough/aspiration, nutritional status (weight/BMI, albumin), need for PPIs, pulmonary function or imaging, and any recurrence or re-intervention at defined intervals (e.g., 3/6/12 months)

 6. Current intraoperative photos (pp. 4–5) would benefit from **annotated arrows/labels** (fistulous tract, diaphragm, pouch, jejunal limb) and consistent terminology in captions. Add scale/context where possible. Please complement intraop images with pre-/post-treatment imaging as above.

 7.  The discussion attributes GBF to “insufficient treatment of early leaks/collections,” yet the case also includes a **post-cholecystectomy subphrenic abscess**, which may have contributed to diaphragmatic erosion. Please strengthen the causal reasoning with a schematic model: proximal staple-line leak → subphrenic abscess → diaphragmatic erosion → broncho-gastric communication; and discuss the influence of **downstream sleeve stenosis/pressure** if present

 Minor Issues:

 Several minor issues detract from the manuscript's clarity and professionalism.

     •    The figures are not sequentially numbered in the text, which can cause confusion for the reader.

     •     Several abbreviations (e.g., GBF, CT, EGOD) are used without being defined on their first use.

     •    The timeline of events could be presented more clearly. A simple table or a more structured narrative would improve readability.

     •    VThe manuscript contains several vague statements, such as “pus can eventually erode through the peripheral diaphragm,” which lack scientific precision.

- Page 3 opens with “ **Obesity is rare**,” which is incorrect and contradicts the following sentence; revise to “ **Obesity is common** … affects ~1 in 8 people worldwide.” Remove duplicated sentences and harmonize global burden statements.  
- Standardize decimals to a period (0.22%, not 0,22%) and ensure percentages are evidence-based (mortality rates, case numbers).
- Correct numerous typographical errors (e.g., “howver,” “wordwide,” “double-pig” → **double pigtail**, “ronchial” → **bronchial**, “EGOD” → **EGD**, “taught” → **taut**, “foradequate” → **for adequate**; “for the Surgery of Surgery” → **International Federation for the Surgery of Obesity and Metabolic Disorders (IFSO)**). Define all acronyms at first use. N.B. Use the new name of IFSO
- Use past tense consistently; standardize procedure names; avoid ambiguous phrases (“prosthesis 3 weeks later”).
- Provide **learning points**: (i) persistent respiratory symptoms after SG warrant GBF work-up; (ii) chronic subphrenic sepsis can erode the diaphragm; (iii) define a pivot point from endoscopic to surgical rescue; (iv) rationale for fistulo-jejunostomy selection.  

Language and Style

 The manuscript would benefit from a thorough language and style revision. The tone is at times overly dramatic (e.g., “from a glimmer of hope to a plight”) for a scientific publication. There are also grammatical errors, including inconsistent tense usage and awkward phrasing (e.g., “Downstream stenosis should be considered as chronic fistulas”). 

 Use **metabolic and bariatric surgery (MBS)**; **sleeve gastrectomy (SG)**; **gastro-bronchial fistula (GBF)**. Avoid inconsistent forms such as “gastrobronchiol fistula,” “gastrothoracic fistula,” or “gastrobronchiolitis fistula” unless defined.  References

 The reference list requires significant attention.

     •     Several references are incomplete. For example, Reference 1 (“Obesity and overweight. 2025”) is not a proper citation, and Reference 8 is listed only as “Reference Source.”

     •    Verification: Not all references could be readily verified. For example, Reference 12 is a French thesis that may not be easily accessible to an international audience.

     •    Missed Opportunities: The authors have missed the opportunity to include several highly relevant and highly cited articles that would have provided a stronger context for their case report. The discussion would be significantly strengthened by incorporating findings from these key papers.

 Additional Recommended References

 The following references should be reviewed by the authors and considered for inclusion in their discussion:

   refer to 1,2,3,4

 References

 refer to 5,6,7

Are enough details provided of any physical examination and diagnostic tests, treatment given and outcomes?

Partly

Is the case presented with sufficient detail to be useful for other practitioners?

No

Is sufficient discussion included of the importance of the findings and their relevance to future understanding of disease processes, diagnosis or treatment?

Partly

Is the background of the case’s history and progression described in sufficient detail?

Partly

Reviewer Expertise:

MBS

I confirm that I have read this submission and believe that I have an appropriate level of expertise to confirm that it is of an acceptable scientific standard, however I have significant reservations, as outlined above.

## References

[1] Rogalski P, et al. (2020). Endoscopic management of leaks and fistulas after bariatric surgery. Prz Gastroenterol. 2020;15(4):356-362.

[2] Campos JM, et al. (2011). Gastrobronchial fistula after sleeve gastrectomy and gastric bypass: endoscopic management and prevention. Obes Surg. 2011;21(9):1339-1345. 10.1007/s11695-011-0444-821643779

[3] Silva LB, et al. (2015). Gastrobronchial fistula in sleeve gastrectomy and Roux-en-Y gastric bypass—a systematic review. Obes Surg. 2015;25(10):1927-1932. 10.1007/s11695-015-1822-426224373

[4] Hany M, et al. (2025). Combined Laparoscopic and Thoracoscopic Management of Gastrobronchial Fistula Developed Early After Laparoscopic Sleeve Gastrectomy: A Video Presentation. Obes Surg. 2025;35(9):3952. 10.1007/s11695-025-08112-zPMC1245745940819024

[5] : Combined Laparoscopic and Thoracoscopic Management of Gastrobronchial Fistula Developed Early After Laparoscopic Sleeve Gastrectomy: A Video Presentation. *Obesity Surgery* .2025;35(9) : 10.1007/s11695-025-08051-9 3949-3951 10.1007/s11695-025-08051-9 PMC1245746240660063

[6] : Gastric Fistula in the Chest After Sleeve Gastrectomy: a Systematic Review of Diagnostic and Treatment Options. *Obesity Surgery* .2021;31(1) : 10.1007/s11695-020-05078-y 357-369 10.1007/s11695-020-05078-y 33123868

[7] Mahapatra S, et al. S4034 Successful Closure of a Gastro-Bronchial Fistula… Am J Gastroenterol. 2024;119(10S):pS2425. doi:10.14309/01.ajg.0000959487.33333.f3.
